# Association between health literacy and the time to first cigarette among daily smokers in Zhejiang Province, China

**DOI:** 10.3389/fpubh.2025.1620838

**Published:** 2025-11-06

**Authors:** Xiujing Hu, Dingming Yao, Heni Chen, Qiaohong Lv, Xiaotong Yan, Yusui Zhao, Xuehai Zhang, Yue Xu

**Affiliations:** Department of Health Education, Zhejiang Provincial Center for Disease Control and Prevention, Hangzhou, China

**Keywords:** health literacy, time to first cigarette, nicotine dependence, daily smokers, threshold effect

## Abstract

**Background:**

Nicotine dependence significantly impedes smoking cessation efforts, yet limited research has explored its relationship with health literacy in the Chinese context. This study aimed to investigate the association between health literacy and nicotine dependence among daily smokers in Zhejiang Province, China, with particular focus on potential threshold effects.

**Methods:**

We conducted a cross-sectional study involving 3,235 daily smokers (99.23% male) from the 2022 Chinese Health Literacy Survey in Zhejiang Province. Health literacy was assessed using a validated Chinese health literacy scale (0–66 points), while nicotine dependence was measured by time to first cigarette (TTFC ≤ 30 min indicating high dependence). Multivariable logistic regression and threshold effect analysis were conducted to examine the relationship between health literacy levels and nicotine dependence.

**Results:**

Health literacy was significantly inversely associated with high nicotine dependence across all models (fully-adjusted *OR* = 0.99 per point increase, 95%*CI*: 0.98–0.99, *p* < 0.001). A clear threshold effect was observed at 53 points (the standard for adequate health literacy), with individuals scoring ≥53 having 34% lower odds of high nicotine dependence compared to those with below basic literacy (*OR* = 0.66, 95%*CI*: 0.50–0.87, *p* = 0.003). A significant dose–response relationship was evident across health literacy categories (*P* for trend <0.001), with protective effects emerging at intermediate literacy levels (40 ~ 52 points) and strengthening at adequate levels (53 ~ 66 points).

**Conclusion:**

Health literacy exhibits an independent, protective association against nicotine dependence among daily smokers in this predominantly male sample, with effects becoming pronounced above the adequacy threshold. These findings suggest that integrating tobacco control objectives within China’s existing health literacy promotion framework may enhance smoking cessation efforts and reduce nicotine dependence, particularly in regions like Zhejiang Province that continue to face high male smoking prevalence despite active tobacco control policies.

## 1 Introduction

Tobacco use remains one of the largest public health threats globally, with over 8 million deaths annually due to tobacco-related diseases (1), placing a substantial burden on both economic and healthcare systems. In China, over 300 million people smoke, and tobacco-related diseases account for more than 1 million deaths annually and are projected to reach 2 million by 2030 (2). Epidemiological data indicate that 49.7% of Chinese current smokers exhibit severe nicotine dependence (3), creating significant challenges for smoking cessation initiatives and public health interventions.

Nicotine, the principal psychoactive component in tobacco products, functions as the key mediator of addiction through its interactions with neurochemical pathways (4). Nicotine dependence plays a pivotal role in shaping smoking behavior and influencing cessation outcomes, with highly dependent smokers facing greater challenges in quitting and experiencing higher relapse rates (5, 6). The Fagerström Test for Nicotine Dependence (FTND) has been validated as a standardized instrument for nicotine dependence assessment (7). Time to First Cigarette (TTFC), the initial component of FTND, quantifies the time interval between awakening and the first cigarette consumption. TTFC serves as a reliable indicator of physiological nicotine dependence severity and correlates significantly with cessation difficulty, relapse probability, and smoking-related health risks (8–10). Additionally, genetic studies have supported the utility of TTFC as an independent measure of nicotine dependence. A previous genetic study on nicotine dependence has demonstrated associations between FMO3 gene polymorphisms and extended TTFC duration, while these genetic variations showed no correlation with daily cigarette consumption (11). This independent nature of TTFC highlights its value as a key indicator in nicotine dependence research (12).

Health literacy (HL) has emerged as a critical research focus in public health studies. The concept encompasses an individual’s abilities to obtain, process, and utilize health-related information (13). Health literacy demonstrates significant associations with both health-related decision-making processes and measurable health outcomes (14–16). Enhanced health literacy may facilitate individuals’ comprehension of smoking-related health risks, potentially modifying their smoking behavior patterns (17, 18).

Despite its recognized importance in health promotion, the specific relationship between health literacy and nicotine dependence remains inadequately explored, particularly regarding physiological indicators such as TTFC. As a robust marker of nicotine dependence severity, TTFC offers a unique opportunity to investigate how cognitive factors (such as health literacy) might interact with the physiological and behavioral aspects of addiction. Understanding these connections could reveal novel pathways for developing more effective, targeted smoking cessation interventions, especially for highly dependent smokers in high-prevalence settings such as China.

This study aims to investigate the association between health literacy and TTFC among daily smokers in China. We hypothesize that higher health literacy levels are linked to a longer TTFC duration, indicating a lower level of nicotine dependence. The findings will provide empirical evidence for developing targeted smoking cessation interventions and contribute to the broader understanding of health literacy’s role in nicotine dependence.

## 2 Materials and methods

### 2.1 Survey description

This cross-sectional study utilized data from the 2022 Chinese Health Literacy Survey (CHLS), which was conducted by the Chinese Center for Health Education using a complex multistage, stratified cluster sampling design. The CHLS study followed the ethical principles of the 1975 Declaration of Helsinki and was approved by the National Health Commission of China. In Zhejiang Province, the survey was implemented by the Zhejiang Provincial Center for Disease Control and Prevention (CDC) under standardized protocols and received ethical approval from its Ethics Committee (approval number: 2022–027-01).

The sampling in Zhejiang Province was proceeded in five stages: (1) 30 counties were randomly selected across the province; (2) within each county, 4 townships were randomly selected; (3) within each township, 2 residential blocks were chosen; (4) a complete list of household addresses was compiled for each block, and 80 households were randomly selected; (5) finally, one participant per household was chosen using the Kish grid method, ensuring equal representation and minimizing selection bias. Informed consents were obtained from all survey participants.

Smoking status was assessed with the question “Do you currently smoke tobacco products?” In accordance with the criteria set by the Tobacco Control Office of the Chinese Center for Disease Control and Prevention (China CDC), participants who answered “Yes, I smoke every day” were categorized as daily smokers, while those who answered “Yes, but not every day” were categorized as occasional smokers. Only daily smokers were included in the present analysis to focus on individuals with established smoking patterns.

**Figure 1:**
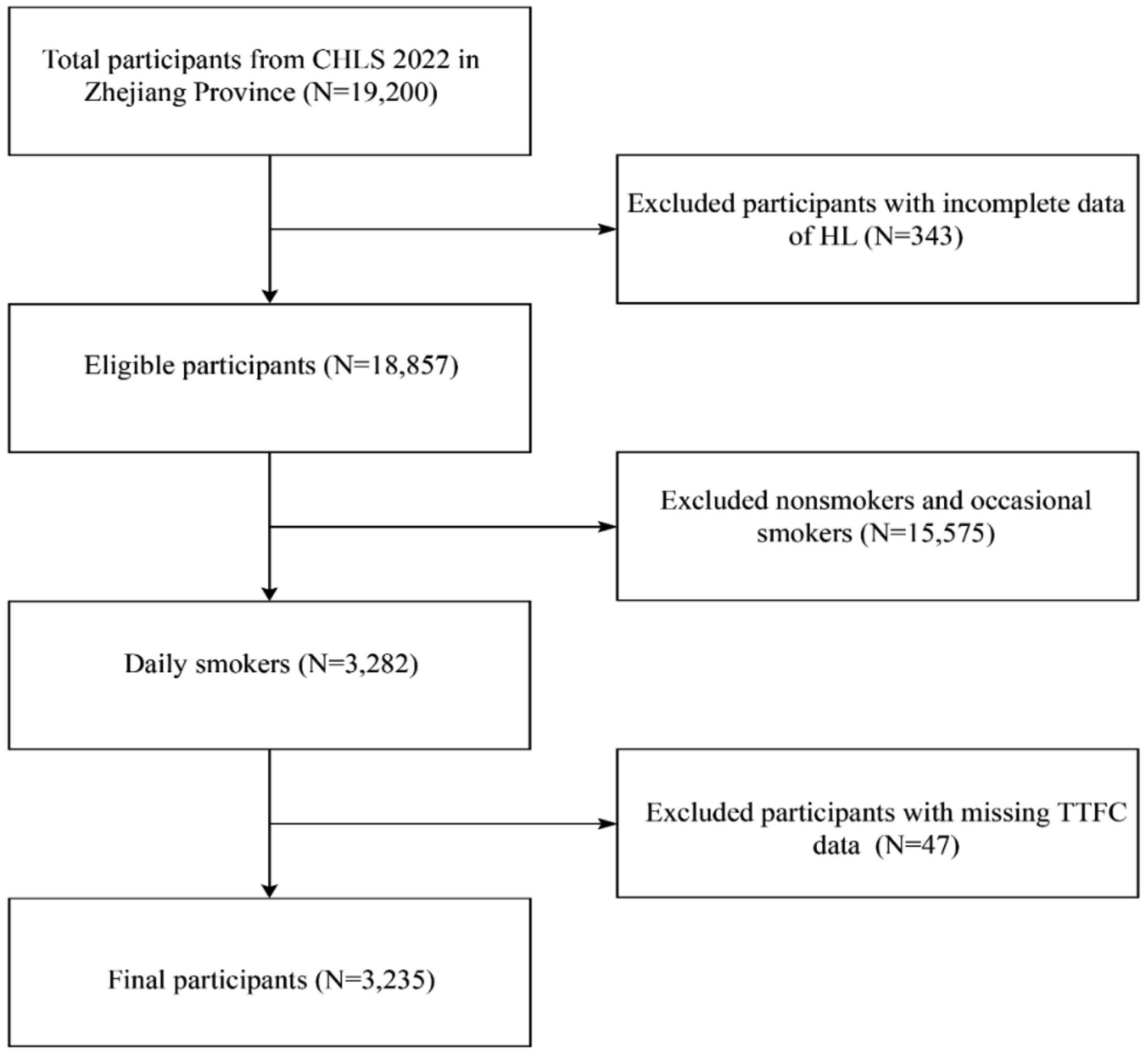
Flowchart of the sample selection process.

Of the 19,200 participants (aged 15–69 years) initially enrolled in Zhejiang province, 3,235 eligible subjects were included in the final analysis after excluding non-smokers and occasional smokers (*n* = 15,575), participants with missing health literacy data (*n* = 343), and those lacking TTFC information (*n* = 47). Details of the participant selection process are illustrated in Figure 1.

### 2.2 Assessment of health literacy

Health literacy was assessed using the Chinese Health Literacy Scale for Residents, developed by the Chinese Center for Health Education. This validated instrument comprises 50 items (total score: 66 points) across six dimensions: scientific health concepts, health information, prevention and treatment of infectious diseases, prevention and treatment of chronic diseases, safety and first aid, and basic medical care (19, 20). The scale has strong internal consistency and split-half reliability (the Cronbach’s *α* coefficient was 0.909 and the split-half correlation coefficient was 0.829) (19). Based on established criteria (19), participants can be categorized into 4 levels of health literacy: below basic (0 ~ 26 points), basic (27 ~ 39 points), intermediate (40 ~ 52 points) and adequate (53 ~ 66 points).

### 2.3 Assessment of TTFC

The TTFC was utilized as a rapid assessment tool for evaluating the degree of tobacco dependence in the study (12). It was measured based on smoker’s answer to the question of “How soon do you smoke your first cigarette after you wake up?,” the response options are within ≤5 min, 6–30 min, 31–60 min, and >60 min of waking (21). Consistent with previous researches, the study further classifies a TTFC ≤ 30 min as indicative of cigarette dependence (22–24).

### 2.4 Assessment of covariates of interest

Sociodemographic variables included age (15–29, 30–44, 45–59, and 60–69 years), sex, educational level (primary school or below, middle school, high school and above), marital status (single, married, separated/divorced/widowed), and urbanicity (urban areas, rural areas).

Health-related variables included chronic conditions and self-rated health. Chronic conditions were ascertained by asking participants whether they had been diagnosed with any of the following conditions by a healthcare professional: hypertension, diabetes, cardiovascular disease, chronic respiratory disease, malignant tumor or other chronic conditions (yes/no). Free-text “other chronic disease” entries were standardized for reporting; terms that clearly matched a named category were aligned to that category to avoid double counting. Symptom-only or non-chronic expressions were excluded. The standardized list with counts is provided in Supplementary Table S1. For the analysis, we calculated the total number of reported conditions for each participant. To capture the overall disease burden, this count was then categorized into three levels: 0, 1, or ≥2. Self-rated health was measured on a five-level scale (excellent, very good, good, fair, and poor).

### 2.5 Statistical analysis

Descriptive statistics for participant characteristics were summarized as means with standard deviations (SD) for continuous variables and as frequencies (n) with proportions (%) for categorical variables. To compare these characteristics across the TTFC categories, one-way analysis of variance (ANOVA) and the chi-square test were utilized for continuous and categorical variables, respectively.

The association between health literacy and high nicotine dependence (TTFC ≤30 min) was examined using a series of multivariable logistic regression models. There were 3 models applied in the study: Model 1 was unadjusted; Model 2 was adjusted for age and sex; Model 3 was adjusted for age, sex, education level, marital status, urbanicity, chronic conditions, and self-rated health, to control for potential confounding factors that might affect the relationship between health literacy and TTFC.

The adequacy of the analytic sample for multivariable modeling was confirmed. In the primary logistic analysis, there were 1,517 events (TTFC ≤30 min). For the fully adjusted model, the events-per-variable (EPV) was approximately 190 (1,517 events for 8 predictors), substantially exceeding the conventional benchmark of 10 and indicating low risk of overfitting with stable estimates (25). Details are provided in Supplementary method S1.

To further explore the potential non-linear relationship between health literacy and high nicotine dependence, smoothed curve fitting (using the penalized spline method) and generalized additive model (GAM) regression were performed. When a non-linear relationship was identified, the inflection point (threshold) was determined using a likelihood ratio test, which compared the goodness-of-fit between a linear model and a two-piecewise linear regression model. Finally, several sensitivity analyses were conducted to assess the robustness of the primary findings. These included using a stricter definition for high nicotine dependence (TTFC≤5 min) and parameterizing health literacy as a categorical variable. Subgroup analyses stratified by each covariate were also performed.

A two-sided *p*-value of < 0.05 was considered statistically significant. All statistical analyses were conducted using EmpowerStats (http://www.empowerstats.com, X&Y Solutions, Inc.) and statistical software packages R (http://www.R-project.org; The R Foundation).

## 3 Results

### 3.1 Baseline characteristics

A total of 3,235 daily smokers were included in this study. Most participants were male (99.23%), with ages predominantly concentrated in the 45–59 years range (49.49%). The mean health literacy score (mean ± SD) was 41.73 ± 12.80, revealing significant differences among the various TTFC groups (*p* < 0.001). Participants with TTFC > 60 min had the highest score (43.18 ± 13.08). The proportion of participants classified as having adequate health literacy was 22.07%, which increased with longer TTFC durations. Furthermore, statistically significant differences were observed across sex, age, education level, marital status, urbanicity, chronic conditions, and self-rated health (all *p* < 0.05), as detailed in Table 1.

**Table 1:** Basic characteristics of participants by TTFC.

Characteristics	Total, *N*(%)	TTFC, *N*(%)				*P*-value
		≤5 min (*n* = 600)	6–30 min (*n* = 917)	31–60 min (*n* = 813)	>60 min (*n* = 905)	
Sex						0.038
Male	3,210 (99.23)	591 (98.50)	915 (99.78)	805 (99.02)	899 (99.34)	
Female	25 (0.77)	9 (1.50)	2 (0.22)	8 (0.98)	6 (0.66)	
Age (years)						0.003
15 ~ 29 years	98 (3.03)	13 (2.17)	29 (3.16)	23 (2.83)	33 (3.65)	
30 ~ 44 years	622 (19.23)	89 (14.83)	186 (20.28)	163 (20.05)	184 (20.33)	
45 ~ 59 years	1,601 (49.49)	286 (47.67)	459 (50.05)	418 (51.41)	438 (48.40)	
60 ~ 69 years	914 (28.25)	212 (35.33)	243 (26.50)	209 (25.71)	250 (27.62)	
Education level						<0.001
Primary school or below	931 (28.78)	228 (38.00)	263 (28.68)	212 (26.08)	228 (25.19)	
Middle school	1,373 (42.44)	257 (42.83)	420 (45.80)	335 (41.21)	361 (39.89)	
High school and above	931 (28.78)	115 (19.17)	234 (25.52)	266 (32.72)	316 (34.92)	
Marital status						<0.001
Single	239 (7.39)	56 (9.33)	71 (7.74)	49 (6.03)	63 (6.96)	
Married	2,695 (83.31)	457 (76.17)	759 (82.77)	694 (85.36)	785 (86.74)	
Separated/Divorced/Widowed	301 (9.30)	87 (14.50)	87 (9.49)	70 (8.61)	57 (6.30)	
Urbanicity						0.022
Urban areas	1,373 (42.44)	242 (40.33)	359 (39.15)	369 (45.39)	403 (44.53)	
Rural areas	1862 (57.56)	358 (59.67)	558 (60.85)	444 (54.61)	502 (55.47)	
Chronic conditions						0.023
None	2,224 (68.75)	382 (63.67)	641 (69.90)	572 (70.36)	629 (69.50)	
1	822 (25.41)	174 (29.00)	235 (25.63)	199 (24.48)	214 (23.65)	
≥2	189 (5.84)	44 (7.33)	41 (4.47)	42 (5.17)	62 (6.85)	
Self-rated health						0.048
Poor/fair	125 (3.86)	35 (5.83)	30 (3.27)	30 (3.69)	30 (3.31)	
Good	960 (29.68)	196 (32.67)	264 (28.79)	239 (29.40)	261 (28.84)	
Very good/excellent	2,150 (66.46)	369 (61.50)	623 (67.94)	544 (66.91)	614 (67.85)	
Health literacy score, mean ± SD	41.73 ± 12.80	37.85 ± 13.17	41.57 ± 12.41	43.17 ± 12.04	43.18 ± 13.08	<0.001
Health literacy levels						<0.001
Below basic (0–26)	449 (13.88)	131 (21.83)	119 (12.98)	81 (9.96)	118 (13.04)	
Basic (27–39)	816 (25.22)	186 (31.00)	241 (26.28)	191 (23.49)	198 (21.88)	
Intermediate (40–52)	1,256 (38.83)	194 (32.33)	374 (40.79)	348 (42.80)	340 (37.57)	
Adequate (53–66)	714 (22.07)	89 (14.83)	183 (19.96)	193 (23.74)	249 (27.51)	

### 3.2 Association between health literacy and TTFC

The associations between high nicotine dependence (defined as TTFC ≤ 30 min) and health literacy levels are presented in Table 2. In the fully adjusted model (Model 3), which controlled for age, sex, education level, marital status, urbanicity, chronic conditions, and self-rated health, a higher health literacy score remained significantly associated with lower odds of high nicotine dependence. Specifically, each 1-point increase in the health literacy score was associated with a 1% reduction in the odds of having a TTFC ≤ 30 min (*OR* = 0.99, 95%CI: 0.98–0.99, *p* < 0.001). Compared to the below basic level, both intermediate (*OR* = 0.73, 95%*CI*: 0.58–0.93, *p* = 0.011) and adequate (*OR* = 0.66, 95%*CI*: 0.50–0.87, *p* = 0.003) health literacy levels were significantly associated with a reduced risk of high nicotine dependence. Furthermore, a significant trend was observed (*P* for trend<0.001), indicating a decreasing odds of high nicotine dependence as health literacy increases.

**Table 2:** The associations between health literacy and TTFC ≤ 30 min.

Variables	Model 1		Model 2		Model3	
	OR(95% CI)	*P*	OR(95% CI)	*P*	OR(95% CI)	*P*
Health literacy Score	0.98 (0.98, 0.99)	<0.001	0.98 (0.98, 0.99)	<0.001	0.99 (0.98, 0.99)	<0.001
Health literacy levels						
Below basic (0–26)	Reference		Reference		Reference	
Basic (27–39)	0.87 (0.69, 1.10)	0.253	0.88 (0.69, 1.11)	0.266	0.93 (0.72, 1.20)	0.573
Intermediate (40–52)	0.66 (0.53, 0.82)	<0.001	0.66 (0.53, 0.83)	<0.001	0.73 (0.58, 0.93)	0.011
Adequate (53–66)	0.49 (0.39, 0.62)	<0.001	0.49 (0.39, 0.63)	<0.001	0.66 (0.50, 0.87)	0.003
*P* for trend		<0.001		<0.001		<0.001

Model 1: Covariates were not adjusted at all.

Model 2: Adjusted for age and sex.

Model 3: Adjusted for age, sex, education level, marital status, urbanicity, chronic conditions, self-rated health.

### 3.3 Subgroup analysis

Subgroup analyses were conducted to examine the consistency of the association across different strata. As shown in Table 3, no significant interactions were observed across all subgroups (all *P* for interaction > 0.05), indicating that the inverse relationship between health literacy and high nicotine dependence was generally consistent. Consistent with this finding, the inverse relationship remained statistically significant in several specific subgroups. Notably, this association was significant among participants aged 30–44 years (*OR* = 0.98, 95%*CI*: 0.96–1.00, *p* = 0.015) and 60–69 years (*OR* = 0.98, 95%*CI*: 0.97–0.99, *p* < 0.001), those with high school education and above (*OR* = 0.97, 95%*CI*: 0.96–0.99, *p* < 0.001), rural residents (*OR* = 0.98, 95%*CI*: 0.98–0.99, *p* < 0.001), and individuals with good (*OR* = 0.98, 95%*CI*: 0.97–0.99, *p* = 0.002) or very good/excellent self-rated health (*OR* = 0.99, 95%*CI*: 0.98–1.00, *p* = 0.019).

**Table 3:** Subgroups analyses of the effect of health literacy on TTFC ≤ 30 min.

Subgroups	OR (95%CI)	*P*-value	*P* for interaction
Age			0.065
15 ~ 29 years	1.01 (0.96, 1.05)	0.818	
30 ~ 44 years	0.98 (0.96, 1.00)	0.015	
45 ~ 59 years	1.00 (0.99, 1.00)	0.297	
60 ~ 69 years	0.98 (0.97, 0.99)	<0.001	
Education level			0.054
Primary school or below	0.99 (0.98, 1.00)	0.102	
Middle school	0.99 (0.98, 1.00)	0.138	
High school and above	0.97 (0.96, 0.99)	<0.001	
Marital status			0.772
Single	0.99 (0.97, 1.01)	0.298	
Married	0.99 (0.98, 1.00)	0.002	
Separated/Divorced/Widowed	0.98 (0.96, 1.00)	0.050	
Urbanicity			0.068
Urban areas	0.99 (0.99, 1.00)	0.235	
Rural areas	0.98 (0.98, 0.99)	<0.001	
Chronic conditions			0.355
None	0.99 (0.98, 1.00)	0.001	
1	0.99 (0.98, 1.00)	0.040	
≥2	0.97 (0.95, 1.00)	0.019	
Self-rated health			0.483
Poor/fair	0.99 (0.96, 1.02)	0.372	
Good	0.98 (0.97, 0.99)	0.002	
Very good/excellent	0.99 (0.98, 1.00)	0.019	

### 3.4 Threshold effect analysis

**Figure 2:**
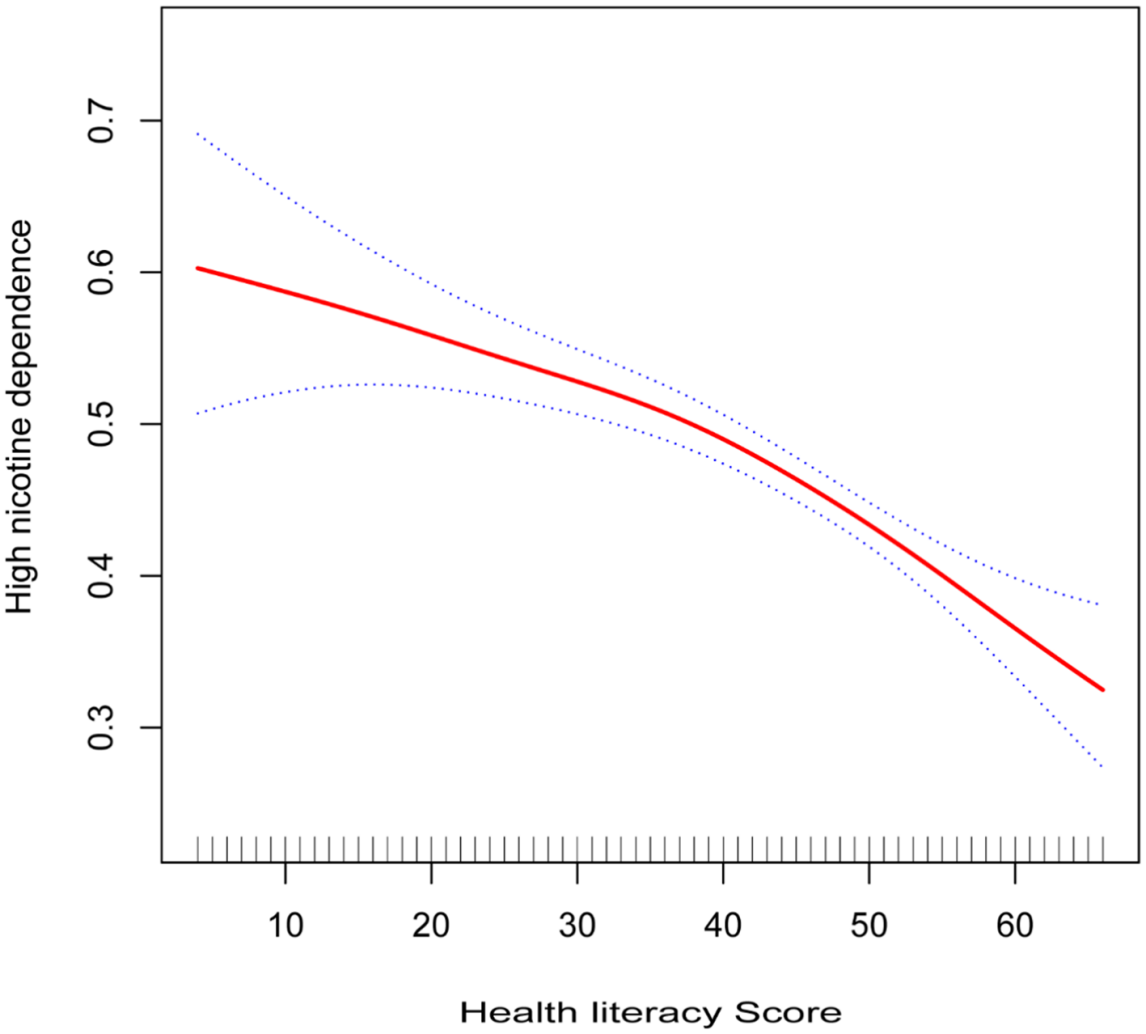
Smooth curve fitting for health literacy score and nicotine dependence.

As illustrated in Figure 2, the results of the smoothed curve fitting suggest a potential non-linear relationship between health literacy scores and high nicotine dependence. Further analyses using a two-piecewise linear regression model, with results presented in Table 4, identified an inflection point at a health literacy score of approximately 53. Before the inflection point, each 1 point increase in health literacy was associated with a 1% reduction in the odds of high nicotine dependence (*OR* = 0.99, 95%*CI*: 0.98–0.99, *p* < 0.001). Beyond the inflection point, this protective association became more pronounced, with each 1 point increase associated with a 6% decrease in the odds (*OR* = 0.94, 95%*CI*: 0.91–0.98, *p* = 0.001). The likelihood ratio test confirmed that the two-piecewise model provided a significantly better fit than the linear model (*p* = 0.025). These findings are primarily applicable to male daily smokers, given the small female representation in the sample.

**Table 4:** Threshold effect analysis of health literacy score on high nicotine dependence.

Health literacy score	Univariate linear regression OR (95%CI)	Two-piecewise linear regression OR (95%CI)	Logarithmic likelihood ratio test *P*-value
<53	0.98 (0.98, 0.99)	0.99 (0.98, 0.99) *P* < 0.001	0.025
≥53		0.94 (0.91, 0.98) *P* = 0.001	

### 3.5 Sensitivity analysis

To assess robustness, we repeated the analysis using a stricter definition of high nicotine dependence (TTFC ≤ 5 min). The results from the fully adjusted logistic regression model showed that the significant inverse association persisted. Even with this stricter cutoff, each 1-point increase in the health literacy score was associated with a 2% decrease in the odds of extremely high nicotine dependence (*OR* = 0.98, 95%*CI*: 0.97–0.99, *p* < 0.001). These findings indicate that our results are not sensitive to the choice of TTFC cutoff. Full estimates and model details are provided in Supplementary Table S2.

## 4 Discussion

This study provides novel evidence of the complex relationship between health literacy and nicotine dependence among daily smokers in China. By examining time to first cigarette (TTFC ≤30 min) as an indicator of high nicotine dependence, our findings reveal both continuous and threshold effects in this association. In the continuous analysis, each one-point increase in health literacy score was associated with a 1% reduction in high nicotine dependence risk (*OR* = 0.99, 95%*CI*: 0.98–0.99, *p* < 0.001). More importantly, we identified a significant threshold effect at 53 points—the established standard for adequate health literacy in China (19)—with individuals scoring above this threshold showing a substantial 34% lower likelihood of high nicotine dependence compared to those with below basic literacy (*OR* = 0.66, 95%*CI*: 0.50–0.87, *p* = 0.003). Additionally, our analysis revealed a clear dose–response relationship across health literacy categories, with protective effects emerging at intermediate levels and strengthening at adequate levels (*P* for trend<0.001). These associations remained consistent across various demographic subgroups, suggesting the generalizability of our findings within the smoking population in Zhejiang Province.

Previous research has extensively examined the association between health literacy and smoking behavior. A study based on the CHLS found that health literacy is associated with reduced smoking behavior and enhanced intention to quit smoking (17, 26). For daily smokers, research indicates that individuals with higher levels of health literacy are more likely to quit smoking or reduce their smoking frequency (18), while lower health literacy is associated with greater nicotine dependence. These findings are consistent with the associations observed in our study, further confirming the pivotal role of health literacy in influencing smoking behavior. Our study extends this literature by focusing specifically on nicotine dependence, as measured by TTFC, providing a more granular understanding of how health literacy relates not just to smoking status but to the severity of addiction among daily smokers.

While we did not directly measure the underlying mechanisms, several theoretical frameworks may help explain the observed associations. Individuals with higher health literacy are better positioned to understand smoking-related harms and to navigate health information, which may be linked to more effective smoking-control behaviors. From a cognitive perspective, the Health Belief Model offers a conceptual basis for how health literacy could relate to smoking behavior. Higher health literacy may be associated with greater perceived risk of immediate and long-term smoking harms (18, 27). Such perceptions could plausibly relate to less urgency to smoke after waking, reflected in a longer TTFC.

Similarly, Social Cognitive Theory suggests that self-efficacy may explain our findings. Some studies have found a significant correlation between self-efficacy and health literacy (28, 29). Consequently, health literacy may enhance self-efficacy, which in turn affects smokers’ nicotine dependence. Increased self-efficacy can bolster individuals’ confidence in managing their health, including resisting the urge to smoke, thereby promoting better self-regulation and enabling smokers to postpone the onset of daily smoking. Furthermore, individuals with strong health literacy may be more adept at seeking social support and fostering a social environment that facilitates smoking cessation (30, 31). The environmental feedback from supportive networks may further reinforce healthy behaviors, creating a positive feedback loop that strengthens smokers’ ability to resist immediate nicotine consumption. It is important to note that we did not directly measure risk perception, self-efficacy, or social support in this study. Therefore, these proposed pathways remain hypothetical and warrant investigation in future research specifically designed to examine these potential mediating mechanisms.

This study identified a nonlinear relationship between health literacy and the risk of high nicotine dependence, providing a novel perspective on the influence of health literacy. The protective pattern appeared more pronounced at scores above 53. This finding is close to the established cut-off for adequate health literacy in China (19), suggesting that individuals with higher literacy levels may be better equipped to process and act upon health information, thereby achieving more effective smoking control behaviors. The stronger protective effect observed at higher health literacy levels may reflect the cumulative benefits of improved knowledge, risk perception, and health-related decision-making. While this pattern is consistent with the idea that sufficient literacy supports better appraisal and use of health information, the underlying mechanisms were not directly assessed in our study. These findings underscore the importance of targeting health literacy improvement as part of tobacco control strategies, particularly for individuals with scores below the identified threshold. Enhancing health literacy in this group could yield substantial public health benefits by reducing nicotine dependence. Future research should explore the mechanisms underlying this relationship, including potential mediators such as risk perception, self-efficacy, and access to health resources.

Our findings offer valuable insights for tobacco control in China, where smoking remains deeply embedded in male social interactions and business relationships (32). The identified threshold effect at 53 points—coinciding with China’s standard for adequate health literacy (19)—suggests that the relationship between health literacy and nicotine dependence is not simply linear. This observation may inform smoking cessation strategies in Zhejiang Province, which, despite implementing various tobacco control policies, continues to struggle with high smoking rates among adult males (33).

This work aligns with China’s evolving health promotion approach, which has increasingly recognized health literacy as foundational to public health improvements. With government initiatives now setting specific targets for health literacy improvement (34), an opportunity exists to incorporate tobacco control objectives within this established framework. Rather than creating isolated cessation programs, integrating nicotine dependence content into broader health literacy efforts may yield greater efficiency. This approach leverages existing infrastructure while potentially enhancing smokers’ ability to comprehend and act upon health information. As our results demonstrate protective effects becoming more pronounced above the 53-point threshold, efforts to improve health literacy among smokers may complement conventional measures such as smoking restrictions. For Zhejiang Province, which has prioritized health promotion yet continues to face substantial smoking-related challenges, this health literacy-oriented approach to tobacco control aligns with existing health policy directions while offering a more targeted pathway to address nicotine dependence among daily smokers.

This study has several strengths, including its use of multi-stage stratified random sampling, which enabled a large and representative sample across Zhejiang Province. Furthermore, TTFC, as the most predictive single item in the FTND, is both simple and quick to measure (12). However, several limitations warrant consideration. First, the cross-sectional design precludes causal inference. While higher health literacy was inversely associated with earlier TTFC, reverse causation and residual confounding from unmeasured variables cannot be ruled out. Second, our measures were based on self-report, which may introduce recall and social desirability biases. Although TTFC is considered a reliable and validated measure, and our sensitivity analysis using a stricter cutoff (TTFC ≤ 5 min) confirmed result robustness, reporting bias cannot be entirely eliminated. Third, because 99.23% of participants were men, findings primarily reflect associations among male daily smokers and should not be generalized to women without caution. Future longitudinal studies with objective measures and balanced sex representation are warranted to establish causality and examine potential sex differences.

## 5 Conclusion

In a large, province-wide sample of 3,235 daily smokers in Zhejiang, higher health literacy was inversely associated with high nicotine dependence (TTFC ≤30 min). We observed a clear dose–response relationship and a threshold around 53 points, which is notably close to the national adequacy cut-off. This suggests health literacy as a potentially important and policy-relevant factor in tobacco control strategies. While causality cannot be inferred from self-reported, cross-sectional data, and the predominantly male sample limits generalizability to women, these findings underscore the need for longitudinal and interventional studies to determine whether improving health literacy can delay TTFC and reduce dependence.

## Data Availability

The datasets presented in this article are not readily available because data supporting the findings of this study are available upon reasonable request. Requests to access the datasets should be directed to Yue Xu, yxu@cdc.zj.cn.
