# Supplementary material for: Association between health literacy and the time to first cigarette among daily smokers in Zhejiang Province, China
Source: Front Public Health. 2025 Nov 6;13:1620838. doi: 10.3389/fpubh.2025.1620838 (PMC12631202; doi:10.3389/fpubh.2025.1620838)
Supplement: Supplementary file 1 [file Table_1.docx]

**Supplementary Table S1. Other Chronic Conditions: Standardized List and Counts**

| **System** | **Condition** | **Count (n)** |
| --- | --- | --- |
| Otorhinolaryngology | Chronic rhinitis/pharyngitis/laryngitis | 10 |
| Respiratory | Asthma | 2 |
| Respiratory | Silicosis/pneumoconiosis | 2 |
| Respiratory | Pulmonary bullae | 1 |
| Respiratory | Pulmonary nodule(s) | 1 |
| Endocrine–Metabolic | Hyperuricemia/gout | 16 |
| Endocrine–Metabolic | Dyslipidemia/hyperlipidemia | 12 |
| Endocrine–Metabolic | Thyroid disorders (hyperthyroidism/nodules/postoperative) | 4 |
| Endocrine–Metabolic | Osteoporosis | 1 |
| Gastrointestinal | Gastropathy | 13 |
| Gastrointestinal | Intestinal polyp(s) | 1 |
| Gastrointestinal | Ulcerative colitis | 1 |
| Hepatobiliary | Hepatitis (including chronic hepatitis B) | 14 |
| Hepatobiliary | Liver cirrhosis | 2 |
| Hepatobiliary | Fatty liver | 1 |
| Hepatobiliary | Hepatic cyst | 1 |
| Hepatobiliary | Hepatic hemangioma | 1 |
| Renal/Urologic | Benign prostatic hyperplasia/chronic prostatitis | 2 |
| Renal/Urologic | Chronic cystitis | 1 |
| Renal/Urologic | Chronic kidney disease/uremia | 2 |
| Renal/Urologic | Renal cyst | 1 |
| Rheumatologic | Ankylosing spondylitis | 2 |
| Rheumatologic | Rheumatic disease | 2 |
| Rheumatologic | Rheumatoid arthritis | 2 |
| Rheumatologic | Synovitis | 1 |
| Rheumatologic | Systemic lupus erythematosus | 1 |
| Orthopedic | Cervical spondylosis | 1 |
| Orthopedic | Lumbar disc herniation | 1 |
| Psychiatric | Anxiety disorder | 1 |
| Psychiatric | Depression | 1 |
| Psychiatric | Mental disorder (unspecified) | 1 |
| Psychiatric | Neurasthenia | 1 |
| Vascular (peripheral) | Deep vein thrombosis | 1 |
| Vascular (peripheral) | Varicose veins | 1 |
| Hematologic | Thrombocytopenia | 1 |
| Oncology (unspecified) | Benign tumor/mass | 1 |
| Ophthalmology | Cataract | 1 |

Notes: ‘System’ groupings are for readability and did not enter statistical models.
