# Supplementary material for: Association between health literacy and the time to first cigarette among daily smokers in Zhejiang Province, China
Source: Front Public Health. 2025 Nov 6;13:1620838. doi: 10.3389/fpubh.2025.1620838 (PMC12631202; doi:10.3389/fpubh.2025.1620838)
Supplement: Supplementary file 2 [file Table_2.docx]

**Supplementary Table S2. Sensitivity analyses using TTFC ≤ 5 minutes**

|  | Model 1 | | Model 2 | | Model3 | |
| --- | --- | --- | --- | --- | --- | --- |
|  | *OR*(95% *CI*) | *P* | *OR*(95% *CI*) | *P* | *OR*(95% *CI*) | *P* |
| Health literacy Score | 0.97 (0.97, 0.98) | <0.001 | 0.98 (0.97, 0.98) | <0.001 | 0.98 (0.97, 0.99) | <0.001 |
| Health literacy levels |  |  |  |  |  |  |
| Below basic (0−26) | Reference |  | Reference |  | Reference |  |
| Basic (27−39) | 0.72 (0.55, 0.93) | 0.012 | 0.74 (0.57, 0.97) | 0.026 | 0.83 (0.64, 1.09) | 0.181 |
| Intermediate (40−52) | 0.44 (0.34, 0.57) | <0.001 | 0.48 (0.37, 0.62) | <0.001 | 0.56 (0.43, 0.73) | <0.001 |
| Adequate (53−66) | 0.35 (0.26, 0.47) | <0.001 | 0.38 (0.28, 0.53) | <0.001 | 0.49 (0.35, 0.68) | <0.001 |
| *P* for trend |  | <0.001 |  | <0.001 |  | <0.001 |

Model 1: Covariates were not adjusted at all.

Model 2: Adjusted for age and sex.

Model 3: Adjusted for age, sex, education level, marital status, urbanicity, chronic conditions, self-rated health.
