# Supplementary material for: Association between health literacy and the time to first cigarette among daily smokers in Zhejiang Province, China
Source: Front Public Health. 2025 Nov 6;13:1620838. doi: 10.3389/fpubh.2025.1620838 (PMC12631202; doi:10.3389/fpubh.2025.1620838)
Supplement: Supplementary file 3 [file Supplementary_file_1.docx]

**Supplementary Methods S1. Sample size: parent survey design and adequacy of the smoker sub-sample**

**A. Parent survey: sampling and a-priori size determination**

The parent Zhejiang Health Literacy Survey used multi-stage, stratified cluster sampling to draw a province-representative sample (ages 15-69). The a-priori target size was based on the formula *N* = *deff*×*Z*_α_/_2_^2^×[*P*×(1－*P*)]/*δ*^2^, using the 2021 provincial health-literacy prevalence *P* = 0.3611, *Z* = 1.96 (95% confidence). Relative precision was set at 15%, with an absolute precision of *δ* = 0.3611 × 15% ≈ 0.054, and design effect (deff) = 1.0. The minimum per-stratum sample size was 304; allowing for operational factors, 640 participants were targeted per monitoring point (30 points in total; target = 19,200). In practice, 18,857 valid responses were obtained. Standardized instruments and interviewer training ensured data quality.

**B. Analytic sub-sample and adequacy for modeling**

This study analyzed a subset of 3,235 daily smokers from the parent survey. The primary outcome was high nicotine dependence, defined as TTFC ≤ 30 minutes (E = 1,517 events; 1,718 non-events). For the logistic regression model including health literacy and seven covariates (K = 8), the events-per-variable (EPV) was 1,517 / 8 ≈ 190, well above the conventional threshold of 10 events per variable for stable estimation and minimal overfitting risk (Peduzzi et al., 1996).

The large sample size also ensured high precision in effect estimation, with an adjusted odds ratio (a*OR*) of 0.99 for health literacy (95% *CI*: 0.98-0.99).

**C. Sensitivity analysis**

Under alternative TTFC cut-points (e.g., ≤5 minutes), event counts change modestly; EPV remains well above conventional thresholds in similarly specified models. The inverse association between health literacy and nicotine dependence also persisted under the stricter TTFC definition in fully adjusted models (see Results and Supplementary Table S2).

**Reference**

Peduzzi P, Concato J, Kemper E, Holford TR, Feinstein AR. A simulation study of the number of events per variable in logistic regression analysis. J Clin Epidemiol. (1996) 49:1373-1379. doi: 10.1016/s0895-4356(96)00236-3
